# Metabolic pathways within cTfh subsets and glucose-dependent activation of cTfh17 in SLE and healthy individuals

**DOI:** 10.1172/jci.insight.189858

**Published:** 2025-07-22

**Authors:** Vera Kim, Takaya Misao, Hong Tian, Meggan Mackay, Cynthia Aranow, Sun Jung Kim

**Affiliations:** 1 Center for Autoimmune, Musculoskeletal and Hematopoietic Disease, The Feinstein Institutes for Medical Research, Northwell Health, Manhasset, New York, USA.; 2 Department of Molecular Medicine, Northwell Health-Hofstra School of Medicine, Hofstra University, Hempstead, New York, USA.

**Keywords:** Autoimmunity, Metabolism, Autoimmune diseases, Glucose metabolism, T cells

## Abstract

Cellular metabolism plays a key role in T cell biology. Increased glycolysis and mitochondrial respiration have been identified in CD4^+^ helper T cells from both patients with systemic lupus erythematosus (SLE) and lupus mouse models. Inhibiting this metabolic activity can reduce T cell activation and ameliorate disease symptoms in lupus mice. However, the metabolic differences among circulating follicular helper T (cTfh) cell subsets in patients with SLE versus healthy controls (HCs) have not been thoroughly studied. While the frequencies of cTfh cells and their subsets were similar between patients with SLE and HCs, patients exhibited a higher proportion of activated ICOS^+^ programmed cell death 1–positive cells, which correlated with disease activity. cTfh17 cells from both patients with SLE and HCs demonstrated heightened glycolytic activity and expression of glycolysis-related genes compared with cTfh1 and cTfh2. Glucose deprivation significantly diminished costimulatory molecule expression and cytokine production, including IL-17A, IL-10, IL-2, and TNF-α. Glycolysis inhibition reduced the B cell activation capacity of cTfh17 cells. This glucose dependence was more pronounced in cTfh17 than cTfh2 from patients with SLE, but it similarly affected both cTfh2 and cTfh17 cells from HCs. These findings highlight distinct metabolic dependencies among cTfh subsets and the critical role of glycolysis in cTfh17-mediated B cell activation in SLE.

## Abstract 2

Glucose metabolism is particularly important for cTfh17 activity and may be a therapeutic target for SLE.

## Introduction

Systemic lupus erythematosus (SLE) is a multifaceted autoimmune disorder characterized by chronic inflammation and autoantibody production, leading to multiorgan damage (1). Central to the pathogenesis of SLE are abnormalities in the adaptive immune system, particularly involving T follicular helper (Tfh) cells. Tfh cells are pivotal in driving B cell maturation, germinal center formation, and high-affinity antibody production (2). The pathogenic function of Tfh cells is well accepted and described in animal models of lupus (3, 4). Increased frequency and helper function of Tfh cells are commonly observed in mouse models (5, 6), suggesting that an enhancement of their number or function can predispose to lupus development. An increased frequency of CXCR5^+^ Tfh cells has been observed in the blood of patients with SLE, and the frequency of circulating Tfh (cTfh) cells positively correlates with disease activity (7, 8).

An important observation in human cTfh cells is that they consist of distinct subsets, each with a unique phenotype and function (9). The combination of chemokine receptors CXCR3 and CCR6 defines 3 major cTfh subsets: CXCR3^+^CCR6^–^ (cTfh1), CXCR3^–^CCR6^–^ (cTfh2), and CXCR3^–^CCR6^+^ (cTfh17) (10). The functional alterations of cTfh cells in SLE are not well understood, though several studies have reported an increased frequency of activated ICOS^+^ cTfh cells in active SLE (8), or a shift in the balance of cTfh subsets, with cTfh2 and cTfh17 being overrepresented compared with cTfh1 in adult SLE (11). These findings suggest that cTfh cells in SLE provide enhanced support for B cell activation, as cTfh2 and cTfh17 are efficient B cell helpers (10, 12).

Metabolic reprogramming is increasingly recognized as a fundamental aspect of T cell differentiation and function. T cells adjust their metabolic pathways to meet the energetic and biosynthetic demands of activation, proliferation, and effector functions. Key metabolic processes, including glycolysis, oxidative phosphorylation, and lipid metabolism, influence T cell fate decisions (13). Dysregulation of these pathways in T cells has been implicated in the pathogenesis of SLE (14). Altered cellular metabolism in CD4^+^ T cells has been observed in both patients with SLE and mouse models of lupus (15, 16). This metabolic shift has also been observed in Tfh cells in lupus mouse models, with a focus on the role of glucose metabolism. The inhibition of glucose metabolism prevents the generation and accumulation of Tfh cells in lupus mice but minimally affects Tfh differentiation and humoral immunity following immunization in nonlupus mice (17). mTOR signaling is essential for Tfh differentiation, positively regulating glycolysis primarily mainly through the translation control of mRNAs (18). These studies consistently highlight the importance of cellular metabolism in differentiation of effector T cells and the pathogenesis of SLE. However, the specific metabolic alterations among Tfh subsets and the pathways governing their function with SLE or healthy individuals remain poorly understood.

In this study, we aimed to elucidate the metabolic distinctions among cTfh subsets that contribute to the pathogenic function of Tfh cells in patients with SLE. We also investigated whether these metabolic changes differ between patients with SLE and healthy individuals. Using advanced metabolic profiling techniques and functional assays, we evaluated the metabolic pathways underlying Tfh subset function in the context of SLE. We observed an increase in ICOS^+^ programmed cell death 1–positive (PD-1^+^) activated cTfh cells across all 3 cTfh subsets in SLE, along with enhanced B cell activation and differentiation into antibody-secreting cells, particularly by SLE cTfh17 cells compared with healthy controls (HCs). Activation of cTfh cells induced a robust increase in both mitochondrial function and glucose uptake. Similar levels of activity for both metabolic pathways were observed across cTfh cells from patients with SLE and healthy individuals. While cTfh17 cells from both healthy individuals and those with SLE exhibited elevated glycolysis, the selective dependence on glucose for cTfh17 compared with cTfh2 cells differed between the 2 groups. Inhibition of glycolysis with STF-31 or glucose deprivation prevented the activation of cTfh17 cells and subsequent B cell activation. Our findings reveal that distinct metabolic programs are required by different cTfh subsets and suggest that targeting these pathways could limit the pathogenic function of cTfh17 cells.

## Results

### Increased frequency of activated cTfh cells in patients with SLE.

**Figure 1:**
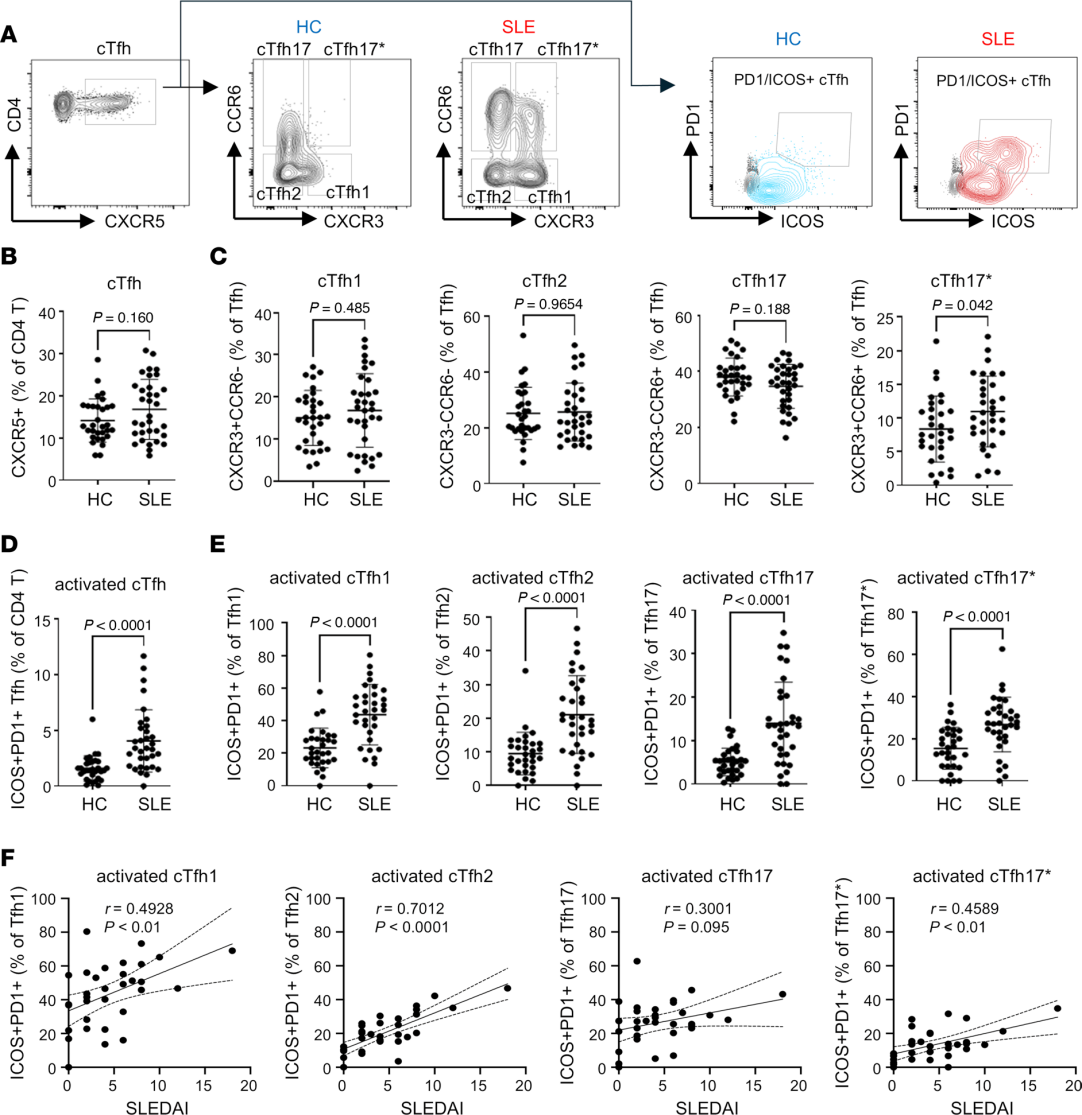
Increased frequency of activated cTfh cells among CD4^+^ T helper cells in the blood of individuals with SLE. PBMCs from HCs and SLE were isolated, and the frequency of cTfh cells and cTfh subsets, and their ICOS/PD-1 expression were investigated using flow cytometry analysis. (**A**) A representative FACS plot illustrating the gating strategy, made from live (fixable viability dye–negative; FVD^–^) singlets. The fluorescence minus 1 of PD-1/ICOS^+^ staining is overlaid in gray. The total percentage of cTfh cells was calculated from the CD4^+^ T cells (**B**), and Tfh subsets were calculated from the total cTfh cells (**C**). The percentage of ICOS^+^PD-1^+^ cTfh cells was determined from the total CD4^+^ T cells (**D**), and ICOS^+^PD-1^+^ cells were determined from each cTfh subset (**E**). Each dot represents an individual sample, and the bar represents mean ± SD. The *P* values were calculated using the nonparametric Mann-Whitney test (*n* = 33). (**F**) Correlation analysis between activated cTfh cell subsets and SLEDAI. Solid line: linear regression, dashed line: 95% confidence interval. Each dot represents an individual sample, and the correlation coefficient and *P* value were calculated using Spearman’s rank correlation method.

CD4^+^ T cells from patients with SLE exhibit several abnormalities that contribute to lupus pathogenesis, including a decrease in naive CD4^+^ T cells, an increase in Tfh cells, and abnormal TCR responsiveness (19). Before assessing the metabolic profiles of cTfh subsets, we characterized the cTfh cells in our cohort of patients with SLE. Patients with SLE and HCs were recruited, and the frequencies of CXCR5^+^CD4^+^ cTfh cells and their subsets (cTfh1: CXCR3^+^CCR6^–^, cTfh2: CXCR3^–^CCR6^–^, cTfh17: CXCR3^–^CCR6^+^) were compared in peripheral blood mononuclear cells (PBMCs). We also evaluated the activation status of cTfh cells and their subsets based on ICOS and PD-1 expression (representative flow images and gating strategies are shown in Figure 1A). There was no difference in the overall frequency of total cTfh cells or cTfh subsets (cTfh1, cTfh2, and cTfh17) between HC and SLE (Figure 1, B and C). In both groups, cTfh17 was the most prevalent subset, followed by cTfh2 and cTfh1. Additionally, we observed a slight but significant expansion of atypical cTfh17 cells (cTfh17*: CXCR3^+^CCR6^+^), which produce both IL-17 and IFN-γ in patients with SLE (20). Although the frequencies of total cTfh or cTfh subsets were similar between patients with SLE and HCs, the percentage of ICOS^+^PD-1^+^ cells was approximately 2-fold higher in total cTfh cells (Figure 1D) and across all cTfh subsets (Figure 1E) in patients with SLE compared with HCs. We also investigated the potential association between activated cTfh subsets and clinical phenotypes. The percentages of activated cTfh1, cTfh2, and cTfh17* were positively correlated with disease activity, as measured by the Systemic Lupus Erythematosus Disease Activity Index (SLEDAI). While activated cTfh17 showed a similar trend, this did not reach statistical significance (Figure 1F). These data verify that cTfh cells exhibit an activated phenotype in SLE, a characteristic observed across all cTfh subsets and positively correlated with disease activity.

### Enhanced glycolysis pathway in cTfh17 versus cTfh1 or cTfh2 subsets.

**Figure 2:**
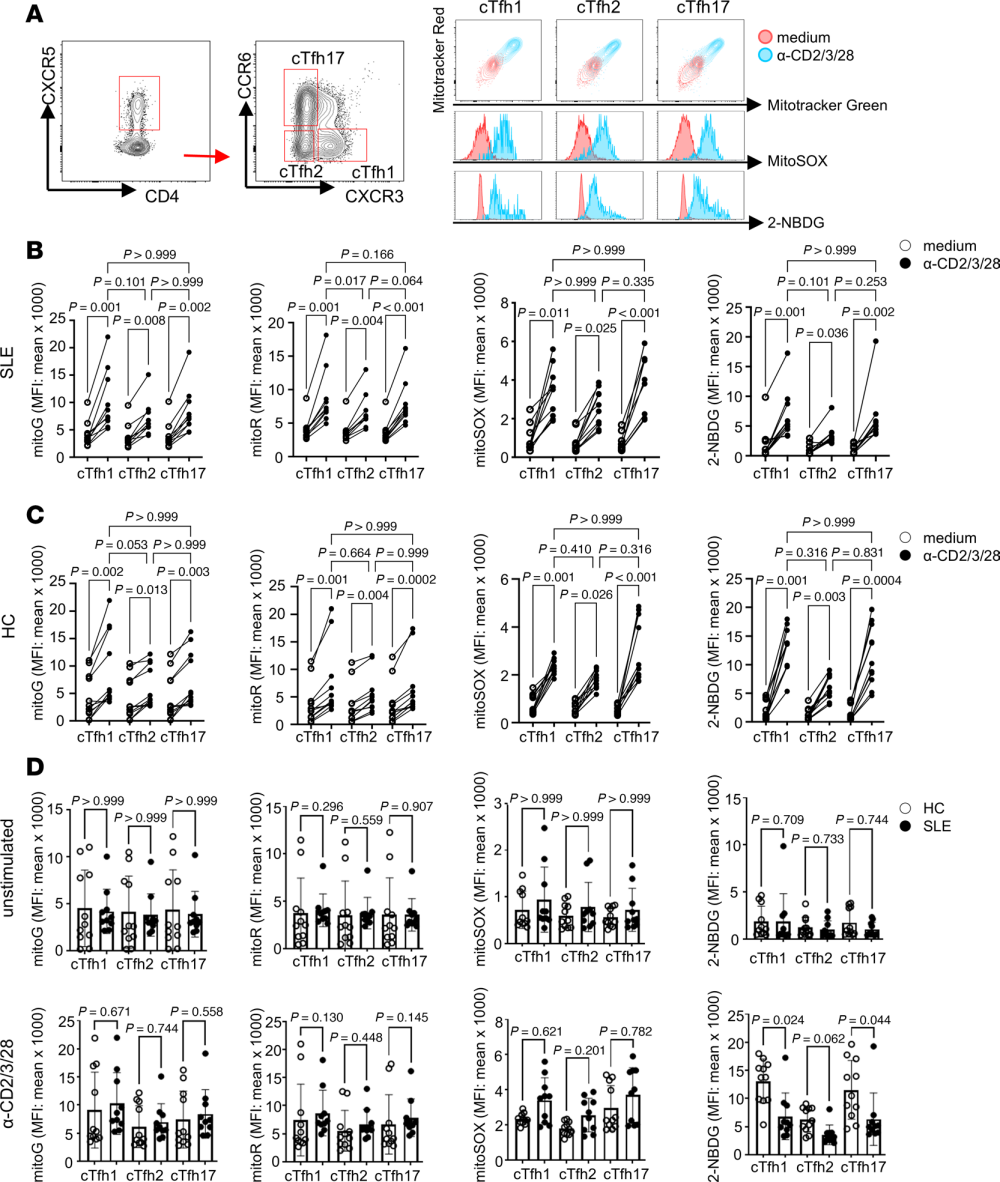
Increased mitochondrial metabolism and glucose uptake in cTfh subsets. Flow cytometry analysis was conducted to evaluate mitochondrial function and glucose uptake in cTfh subsets. (**A**) Representative flow images of Tfh subsets (cTfh1: CXCR3^+^CCR6^–^, cTfh2: CXCR3^–^CCR6^–^, and cTfh17: CXCR3^–^CCR6^+^) stained with MitoTracker Green FM, MitoTracker Deep Red, 2-NBDG, and MitoSOX in unstimulated (medium, red) and stimulated (anti-CD2/3/28, blue) cTfh cells. The MFI for each condition was calculated and graphed for cTfh cells from patients with SLE (**B**) or HCs (**C**). Comparisons of each metabolic dye were grouped by patient group and stimulation condition (**D**) (open circles represent HCs and closed circle represents SLE). Each dot corresponds to an individual sample, and the data shown in **D** are presented as mean ± SD (*n* = 13). Statistical analysis for **B** and **C** used repeated measures Friedman’s test with Dunn’s correction for multiple comparison, while **D** employed Kruskal-Wallis test with Dunn’s correction.

We questioned whether cTfh cell subsets use distinct metabolic pathways to exert their functional activity. Atypical cTfh17 cells are particularly interesting because they are expanded in SLE, but we chose not to study them further because of variability and scarcity in HCs. Previous work by Morel and colleagues reported increased mitochondrial respiration and glycolysis in CD4^+^ T cells from patients with SLE compared with HCs, especially under the activation condition (16). To access the metabolic profile of cTfh subsets, we used met-flow analysis, which allows the study of energy metabolism in small cell populations (21). cTfh subsets were isolated from patients with SLE and HCs and stimulated with anti-CD2/3/28 beads or cultured in medium alone overnight. Mitochondrial metabolism was assessed by measuring mitochondrial mass (Mito Tracker Green), mitochondrial membrane potential (Mito Tracker Red), and mitochondrial reactive oxygen species (ROS) production (MitoSOX). Glucose uptake was measured using the fluorescence-conjugated glucose analog 2-(*N*-(7-Nitrobenz-2-oxa-1,3-diazol-4-yl)Amino)-2-Deoxyglucose (2-NBDG) (representative flow cytometry images are shown in Figure 2A).

Upon activation, all 3 cTfh subsets from patients with SLE exhibited increased mitochondria metabolism, as measured by mitochondrial mass (MitoTracker Green), mitochondrial membrane potential (MitoTracker Red), and mitochondrial ROS production (MitoSOX) (Figure 2B). While activated cTfh1 and cTfh17 cells showed a significant increase or upward trend in mitochondrial membrane potential compared with activated cTfh2 cells, all other measurements demonstrated similar levels in all subsets in both basal and activated status (Figure 2B).

cTfh subsets from HCs displayed similar patterns of mitochondrial function, ROS production, and glucose uptake as those from patients with SLE (Figure 2C). Upon stimulation, all 3 cTfh subsets from HCs showed increased mitochondrial metabolism, ROS production, and glucose uptake. No significant differences were observed across the cTfh subsets from HCs for any of these measurements, except for a trend toward increased mitochondrial mass in activated cTfh1 cells compared with activated cTfh2 cells (Figure 2C).

Finally, we compared these metabolic parameters between HCs and patients with SLE. No differences were observed between unstimulated cTfh cells from the 2 groups (Figure 2D). However, upon activation, cTfh cells from HCs exhibited higher glucose uptake than those from patients with SLE (with cTfh2 cells showing a similar trend). There were no differences in mitochondrial mass, membrane potential, or ROS production between patients with SLE and HCs (Figure 2D).

**Figure 3:**
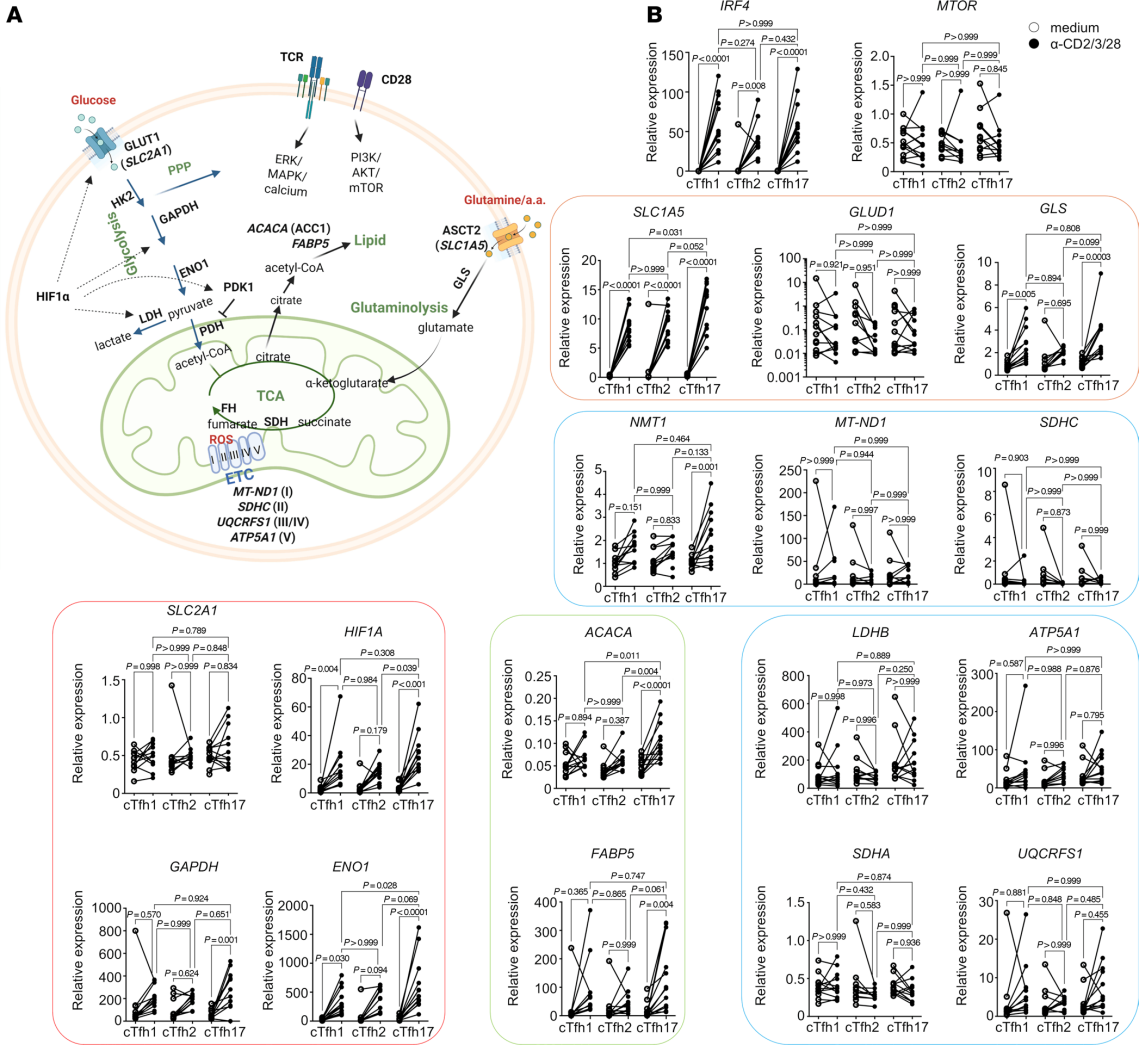
Increased expression of genes in the glycolysis pathway and fatty acid synthesis in cTfh17 cells. (**A**) Diagram of metabolic pathways and enzymes involved in lymphocytes. (**B**) Gene expression levels illustrated in **A** were measured from cTfh subsets isolated from patients with SLE, cultured overnight with or without stimulation beads. After culture, total RNA was extracted, and gene expression was measured by qRT-PCR. Relative expression was calculated using the housekeeping gene *POLR2A* as a reference (*n* = 9). Each dot represents an individual sample, with analysis performed by 1-way ANOVA with Bonferroni’s posttest correction.

We further analyzed gene expression in cTfh subsets from patients with SLE using quantitative reverse transcription (qRT)-PCR, focusing on genes involved in key metabolic pathways (Figure 3A). A gene, *IRF4*, which is related to T cell activation, was upregulated in all 3 cTfh subsets upon activation. Activated cTfh cells showed increased *SLC1A5* and *GLS* expression (but not *GLUD1*) (Figure 3B, orange group), indicating active glutamine metabolism, known to be critical for Th17 cells and disease severity in lupus mice (22). There were no significant changes or patterns in the expression of mitochondria metabolism-related genes (TCA cycle and electron transport chain: *MT-ND1*, *SDHC*, *LDHB*, *ATP5A1*, *SDHA*, and *UQCRFS1*) upon stimulation, except for an increase of *NMT1* in cTfh17 following stimulation (Figure 3B, blue group). Although glucose uptake was increased by stimulation (Figure 2B), glucose transporter (*SLC2A1*, also known as *GLUT1*) mRNA was not increased (Figure 3B). This discrepancy was resolved when we measured protein level of GLUT1. All 3 cTfh subsets showed a marked increase in GLUT1 protein upon stimulation (Supplemental Figure 1A; supplemental material available online with this article; https://doi.org/10.1172/jci.insight.189858DS1). Other genes involved in glycolysis (*HIF1A*, *GAPDH*, *IRF4*, and *ENO1*) were upregulated in activated cTfh1 and cTfh17 but not in cTfh2 (Figure 3B, red group). Additionally, lipid metabolism genes (*ACACA* and *FABP5*) were elevated in activated cTfh17 cells but not in cTfh1 or cTfh2. *ENO1*, *SLC1A5*, and *ACACA* were significantly higher in activated cTfh17 cells compared with cTfh1 and cTfh2. While we observed increased expression of certain genes in cTfh cell subsets from HCs that were also elevated in cTfh cells from patients with SLE, there were ultimately no significant differences in the expression levels of these genes between HCs and patients with SLE (Supplemental Figure 1B).

**Figure 4:**
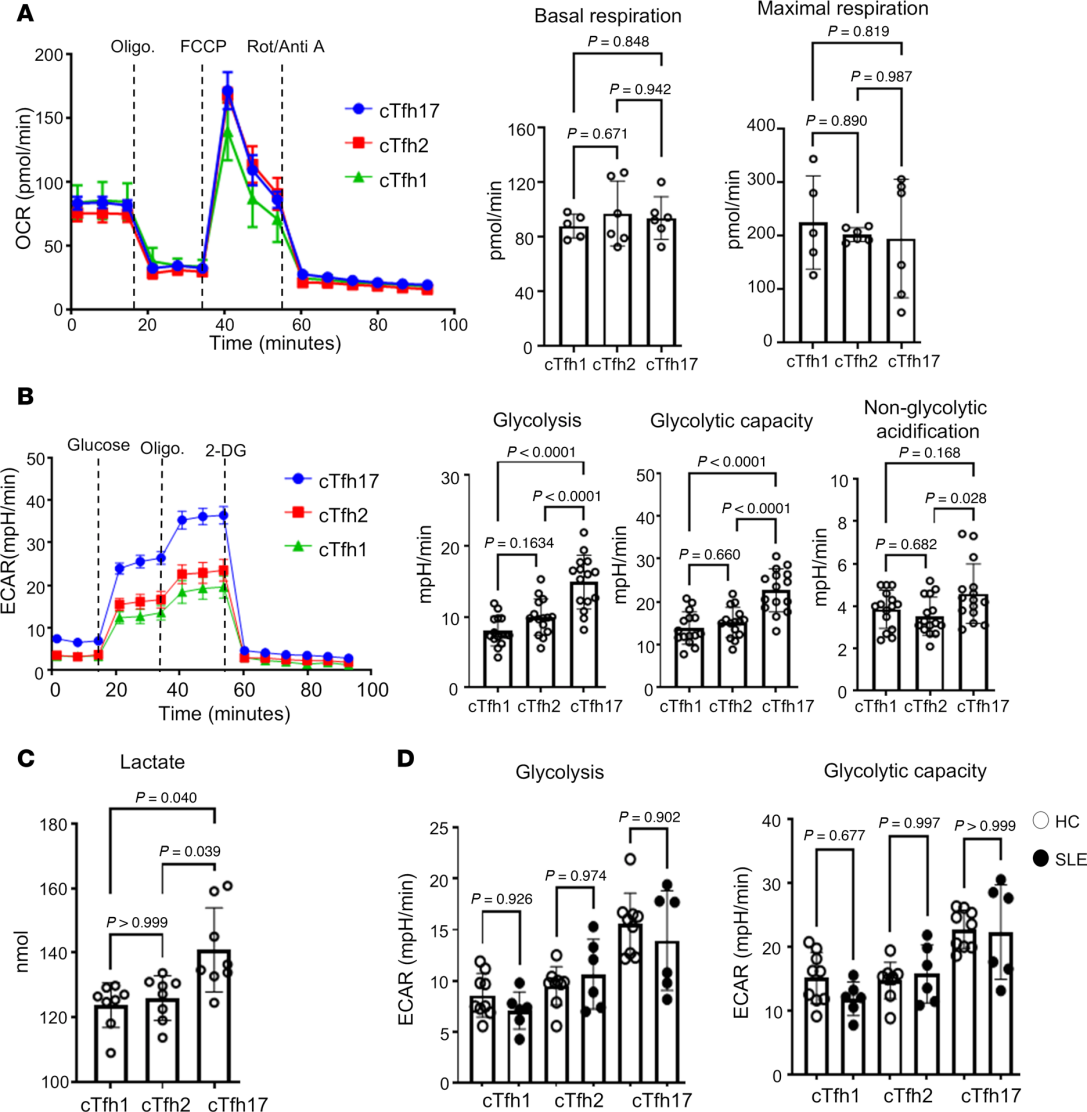
Increased glycolysis in cTfh17 cells. cTfh subsets (cTfh1, cTfh2, and cTfh17) were isolated from patients with SLE and stimulated with anti-CD2/3/28 beads overnight. OCR (**A**) and ECAR (**B**) were measured in each activated cTfh subset. For each assay, a representative analysis panel is shown on the left, with summary graphs on the right. Each dot represents an individual sample, and bar indicates the mean ± SD (*n* = 3). Analysis was conducted using ordinary 1-way ANOVA with Bonferroni’s posttest correction. (**C**) Lactate levels were measured in activated cTfh subsets. (**D**) ECAR was measured in activated cTfh subsets from HCs (open circle) and SLE (closed circle) (*n* = 3). Each dot represents an individual sample and the mean ± SD. Statistical analysis performed using ordinary 1-way ANOVA with Tukey’s correction.

Last, we used a Seahorse analyzer to measure bioenergetics in cTfh subsets (23). We measured the oxygen consumption rate (OCR) and extracellular acidification rate (ECAR) of live T cells under mitochondrial or glycolysis stress conditions. While MitoTracker staining revealed an increase in mitochondrial membrane potential in both cTfh1 and cTfh17cells, no differences in OCR were observed, either at basal or at maximal respiration (Figure 4A). Consistent with gene expression data, activated cTfh17 cells exhibited higher glycolysis (response to glucose) and glycolytic capacity (maximal glycolytic potential) compared with activated cTfh1 and cTfh2 cells (Figure 4B). There was a difference in nonglycolytic acidification between cTfh17 and cTfh2 cells. This increase in glycolysis was validated by elevated lactate secretion from cTfh17 cells compared with cTfh1 or cTfh2 (Figure 4C). While cTfh cells from HCs showed greater glucose uptake than those from patients with SLE, no differences in glycolysis or glycolytic capacity were observed between the cTfh subsets from the 2 groups (Figure 4D). This is consistent with the gene expression data in Figure 3B and Supplemental Figure 1B.

Overall, these data suggest that activated cTfh17 cells have an increased glycolysis compared with cTfh1 and cTfh2, and this elevated glycolysis capacity in cTfh17 cells is subset specific, not dependent on SLE.

### mTOR complex 1 activity is activated more strongly cTfh17 subsets.

**Figure 5:**
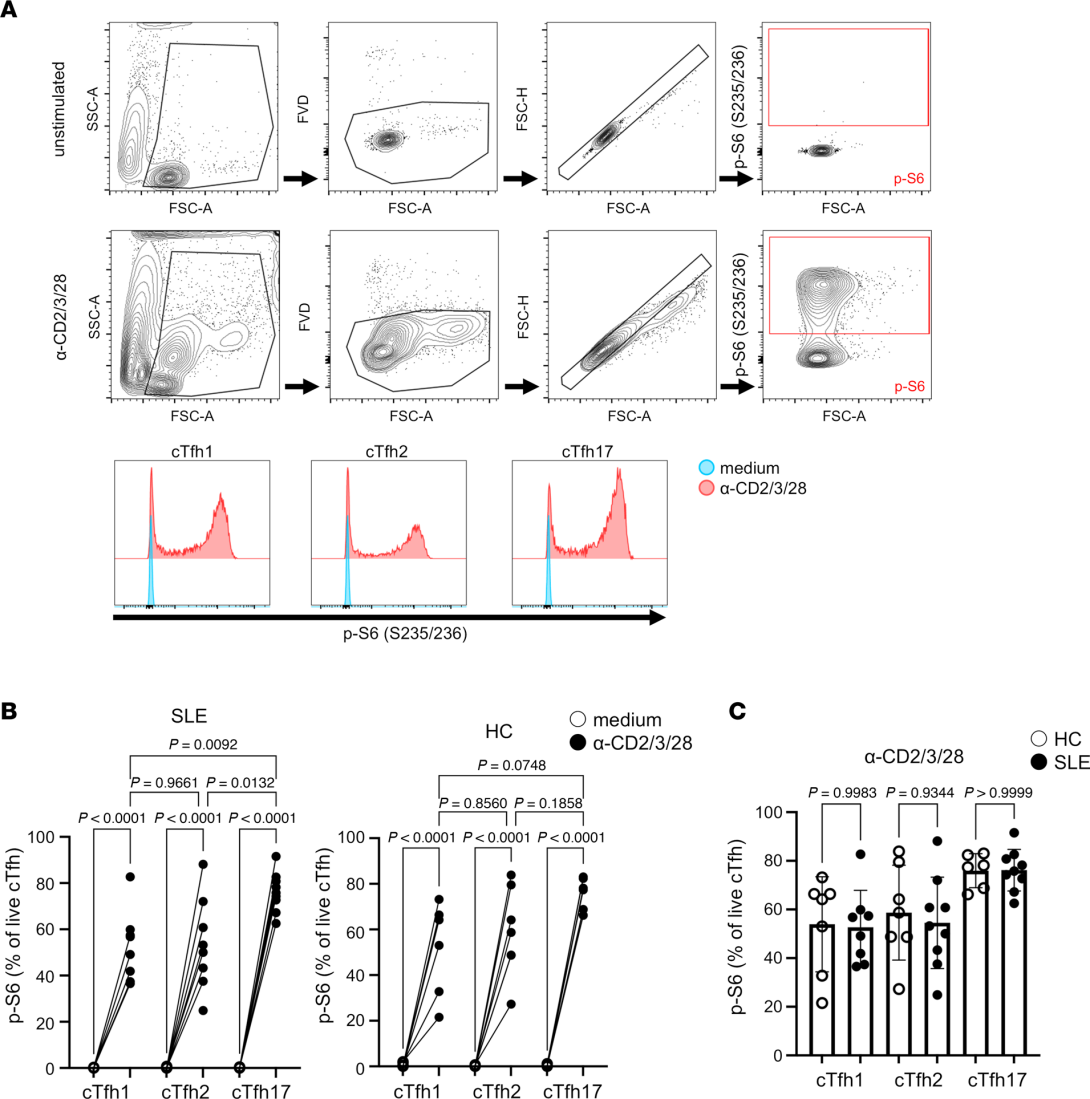
Increased mTOR activity in cTfh17 cells. cTfh subsets were isolated from HCs and patients with SLE and stimulated with anti-CD2/3/28 beads or medium alone for 16 hours. mTOR activity, as assessed by S6 phosphorylation, was measured by measured by flow cytometry. (**A**) Representative gating strategy and flow cytometry plots for each cTfh subset stained for phosphorylated S6 (p-S6) (S235/236). (**B**) Proportion of live p-S6^+^ cells for each subset. Open circles represent unstimulated cells, and closed circles represent stimulated cells. (**C**) Comparison of the frequency of p-S6^+^ cell frequency between HC (open circle) and SLE (closed circle) under stimulated condition. Each dot represents individual samples and bars indicate mean ± SD (*n* = 6–9). Statistical analysis was performed using ANOVA with Šídák’s multiple-comparison test.

Activation of the mTOR pathway is critical for stimulating glycolysis and regulating T cell activation and differentiation (24). We therefore measured mTOR activity in cTfh subsets. Given the absence of increases in *mTOR* mRNA levels (Figure 3B), we assessed mTOR activity by measuring the phosphorylation of ribosomal protein S6 at Ser235/236, a well-established readout for mTOR complex 1 (mTORC1) activity, using flow cytometry (25). As shown in Figure 5A, stimulation with activation beads markedly induced S6 phosphorylation in all 3 cTfh subsets from both in HCs and patients with SLE. In patients with SLE, S6 phosphorylation was significantly higher in cTfh17 cells compared with other subsets (Figure 5B). A similar trend, though not statistically significant, was observed in cTfh17 cells from HCs (Figure 5B). There was no difference in the induction of S6 phosphorylation between HCs and patients with SLE across any of the cTfh subsets (Figure 5C). These results suggest that the mTORC1/S6K/S6 axis is more strongly activated upon stimulation in cTfh17 cells compared with other cTfh subsets in SLE and potentially in HCs as well.

### Higher level of plasma cell induction by cTfh17 from patients with SLE.

The increased activation status of cTfh cells in patients with SLE prompted us to investigate whether cTfh cells from SLE are hyperfunctional. To compare the functional activity of cTfh cells, we performed autologous mixed lymphocyte cultures with memory B cells and each cTfh subset. We focused on the cTfh2 and cTfh17 subsets for the B cell activation assay, as the number of cTfh1 cells was lower, and cTfh1 cells are known to be inefficient helpers, particularly in activating naive B cells (10). Memory B cells were isolated and cultured with either medium alone, a plasma cell (PC) differentiation cocktail (CpG ODN2006, IL-2, IL-10, and IL-21), or cTfh2 or cTfh17 cells for 5 days. After coculture, PCs were identified by their expression of CD27 and CD38, and their isotypes were measured to assess class-switching recombination (Supplemental Figure 2A). As we expected, the PC differentiation cocktail induced a high number of PCs (50%–60% of live B cells) with no significant difference between HCs and patients with SLE (Supplemental Figure 2B). Both cTfh2 and cTfh17 subsets from HC induced PC differentiation (~20%) by cTfh2 and cTfh17, while cTfh17 from patients with SLE induced higher PC differentiation (~40%). SLE cTfh2 cells induced similar percentages of PCs as HC cTfh2 cells (Supplemental Figure 2B). The frequency of class-switched PCs (IgG+ and IgA+) and IgM+ PCs was similar across cTfh2 and cTfh17 subsets from both HC and SLE, indicating that SLE cTfh cells do not preferentially induce class-switching in vitro. However, the amount of IgG and IgA secreted into the supernatant was higher in cultures with SLE cTfh17 cells (Supplemental Figure 2C), likely due to the increased number of PCs induced by SLE cTfh17 cells.

### Glucose restriction decreases activation markers’ expression and cytokine production in cTfh cells from SLE and HCs.

Glucose is a key nutrient essential for T cell differentiation and activation (26–28). However, its specific role in Tfh cell activation and function remains incompletely understood. Our gene expression and Seahorse assay data suggest that glucose may be particularly crucial for cTfh17 cell function. To investigate this further, we examined the impact of glucose deprivation on cTfh17 activation and effector function. We isolated cTfh17 and cTfh2 cells from both patients with SLE and HCs and stimulated them in either complete or glucose-free media. We then measured activation status and cytokine production.

**Figure 6:**
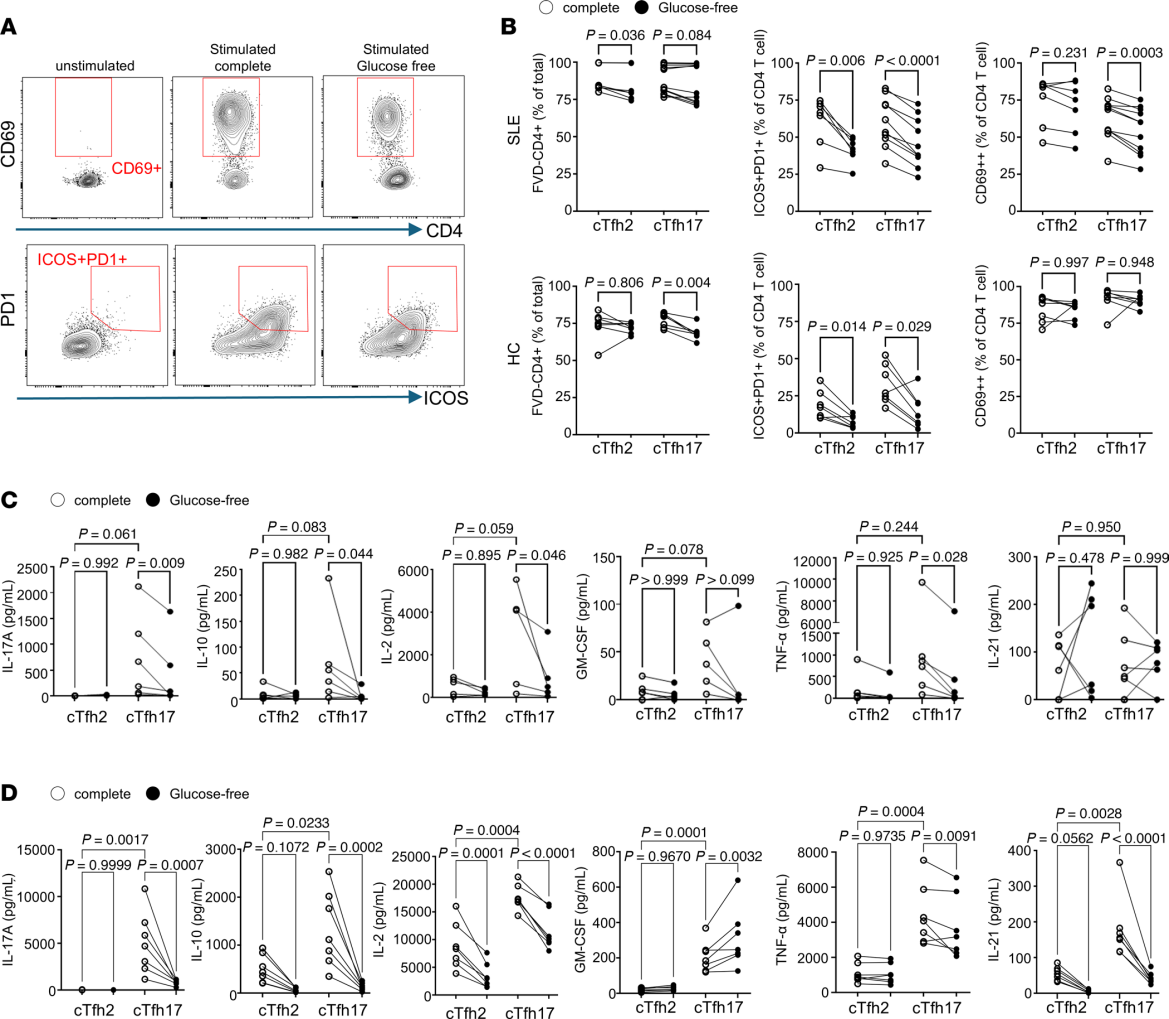
Glucose requirement for optimal activation and cytokine production in cTfh2 and cTfh17. cTfh2 and cTfh17 cells were isolated from patients with SLE and HCs and cultured overnight with or without anti-CD2/3/28 beads in either complete or glucose-free medium. Following stimulation, cell activation and viability were assessed by flow cytometry, and supernatants were collected for cytokine analysis. (**A**) Representative gating strategy for activation markers on cTfh cells. (**B**) Summary graphs of viability and the percentage of CD69^+^ or ICOS/PD-1^+^ cTfh cells in HCs (bottom row) and patients with SLE (upper row). Cytokine levels in the collected supernatants of cTfh cells from SLE (**C**) and HCs (**D**) were measured by Meso Scale Discovery (MSD) multiplex assay. Open circles represent complete medium, and closed circles represent glucose-free conditions (*n* = 6). Each dot represents an individual sample, with statistical analysis performed by 1-way ANOVA with Bonferroni’s posttest correction.

While glucose deprivation did not affect cell viability during stimulation, it significantly reduced the expression of costimulatory molecules ICOS and PD-1 in both cTfh2 and cTfh17 cells from both patients with SLE and HCs (Figure 6, A and B). Although cTfh17 cells have a higher glycolysis capacity than cTfh2 cells, glucose restriction similarly affected ICOS and PD-1 expression in both subsets. CD69 expression decreased in cTfh2 and cTfh17 cells from patients with SLE but remained unchanged in these subsets from HCs (Figure 6B).

Interestingly, we observed a more specific glucose dependence for cytokine production in cTfh17 cells. In patients with SLE, cTfh17 cells produced higher levels of several cytokines, including IL-17A, IL-10, IL-2, and GM-CSF, compared with cTfh2 cells, and these were all reduced under glucose-free conditions (Figure 6C). Glucose deprivation also reduced TNF-α production in cTfh17 cells. However, IL-21, a key cTfh effector cytokine, was produced at similar levels by both cTfh2 and cTfh17 cells and was unaffected by glucose deprivation. In HCs, while baseline cytokine production differed from that in patients with SLE, IL-17A, IL-10, and IL-2 production by cTfh17 cells was similarly reduced under glucose-free conditions. Furthermore, IL-10 and IL-2 production by cTfh2 cells, which was unaffected in patients with SLE, was decreased in HCs under glucose deprivation. Importantly, IL-21 production, which was unaffected by glucose deprivation in SLE, was strongly suppressed in HCs under glucose-free conditions (Figure 6D). Production of other Th17 family cytokines (IL-17E and IL-17F) and the Th2 family cytokines (IL-4 and IL-13) was also not affected by glucose deprivation in patients with SLE (Supplemental Figure 3). In contrast, IL-4, IL-13, and IFN-γ production was reduced in cTfh2 and cTfh17 cells from HCs but not from SLE (Supplemental Figure 3).

These findings indicate that glucose, or glucose-dependent metabolism, is essential for the activation of both cTfh2 and cTfh17 cells. In patients with SLE, glucose specifically promotes the production of pro-inflammatory cytokines by cTfh17 cells but not cTfh2 cells. However, in HCs, glucose appears to have a broader impact on both cTfh2 and cTfh17. The observed differences in cytokine production between HCs and patients with SLE under glucose-free conditions suggest that glucose dependency is altered in SLE.

### Inhibition of glucose metabolism suppresses cTfh17 cell activation and effector function.

**Figure 7:**
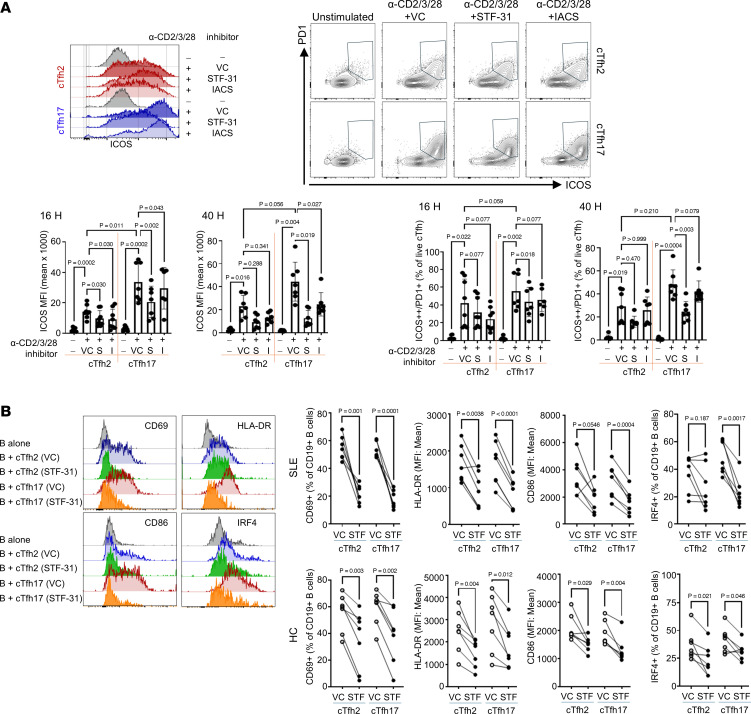
STF-31 pretreatment reduces cTfh17 cell and B cell activation function. (**A**) cTfh2 and cTfh17 cells were isolated from patients with SLE and activated with or without activation beads under various inhibitor conditions (VC, DMSO vehicle control; S, STF-31; I, IACS-010759). After 16 hours and 40 hours, cell viability and activation were assessed by flow cytometry. MFI of ICOS was compared in each cTfh subset (bottom left). The frequency of ICOS^+^PD-1^+^ cTfh2 and cTfh17 cells was quantified from viable cells and graphed (bottom right) (*n* = 6). Each dot represents an individual sample, and the bar represents mean ± SD. (**B**) cTfh2 and cTfh17 cells were isolated from patients with SLE or HCs and pretreated with VC (DMSO) or STF-31 overnight. Cells were washed to remove inhibitor and cocultured with autologous memory B cells and stimulation beads for 2 days. The viability and phenotype of CD19^+^ B cells were analyzed by flow cytometry. Representative overlay flow images are on the left, and summary graphs with paired comparison between VC and STF-31 are on the bottom 2 rows (top right for SLE and bottom right for HCs) (*n* = 5). Each dot represents an individual sample. Statistical analysis performed by ordinary 1-way ANOVA with Bonferroni’s posttest correction.

To verify the essential role of glucose metabolism in cTfh17 cell activation and effector function, we used pharmacological inhibitors. Specifically, we employed STF-31 and IACS-010759, which potently and selectively target the glucose transporter GLUT1, and oxidative phosphorylation complex I, respectively (29, 30). Both inhibitors, when used as single treatments, reduced ICOS and PD-1 expression in activated cTfh17 cells at both 16 and 40 hours. In cTfh2 cells, ICOS and PD-1 expression decreased at 16 hours but not at 40 hours (Figure 7A). While both drugs reduced ICOS expression (Figure 7A), STF-31 more strongly inhibited the activated ICOS^+^PD-1^+^ cTfh17 population (Figure 7A). Notably, CD69 expression was unaffected by either STF-31 or IACS-010759 on both cTfh2 and cTfh17 cells (Supplemental Figure 4).

Next, we assessed the impact of STF-31 pretreatment on the ability of cTfh2 or cTfh17 cells to activate B cells. Memory B cells were cocultured for 40 hours with autologous cTfh2 and cTfh17 cells that had been pretreated with either STF-31 or vehicle control (DMSO). STF-31 pretreatment markedly suppressed memory B cell activation, reducing the expression of CD69, HLA-DR, and CD86 by both cTfh2 and cTfh17 cells (Figure 7B). IRF4 expression in memory B cells, a marker of PC differentiation, was significantly decreased when cocultured with STF-31–pretreated cTfh17 cells but not with STF-31–pretreated cTfh2 from patients with SLE. In HCs, we observed significant decreases in all activation markers (CD69, HLA-DR, and CD86) and IRF4 in B cells cocultured with STF-31–pretreated cTfh2 and cTfh17 (Figure 7B).

In addition to glucose metabolism, cTfh17 cells exhibited higher expression of *SLC1A5* and *GLS1* compared with cTfh1 and cTfh2 cells (Figure 3), suggesting a potential role for glutamine metabolism in cTfh17 activation. Glutamine metabolism has been implicated in the pathogenic function of Th17 cells in both patients with SLE and the MRL/lpr lupus mouse model (22, 31). To investigate this, we treated cTfh17 cells with C968, a glutaminase inhibitor (32). However, rather than reduction, C968 treatment unexpectedly increased the proportion of activated (ICOS^+^PD-1^+^) cTfh17 cells (Supplemental Figure 5).

In summary, cTfh17 cells from patients with SLE require glucose and glucose-dependent metabolic pathways for optimal activation and cytokine production, processes crucial for B cell activation. Inhibiting glucose metabolism in cTfh17 cells effectively suppresses their pathogenic function in SLE. Glucose is also required for optimal activation and cytokine production in cTfh cells from HCs, with this dependence extending to other cTfh subsets, including cTfh2 cells.

## Discussion

cTfh cells encompass subsets associated with Th1, Th2, and Th17 cells (10), advancing our understanding of cTfh diversity and their relationship to other Th subsets. While functional differences among cTfh subsets are frequently reported in SLE, the precise role of each subset remains unclear. Our study showed that activated cTfh17 cells from patients with SLE exhibit an increased B cell activation and antibody production, potentially contributing to pathogenesis in SLE. Metabolically, cTfh17 cells have a higher glycolytic activity than cTfh1 or cTfh2 cells in both HCs and patients with SLE. This increased glucose dependence leads to suppressed costimulatory molecule upregulation and cytokine production under glucose-restricted conditions. In patients with SLE, inhibiting glycolysis with STF-31 significantly reduced the B cell–activating capacity of cTfh17 cells (Figure 7). Moreover, while costimulatory molecule expression depends on glucose in both cTfh2 and cTfh17 cells, decreased cytokine production was observed only in cTfh17, not in cTfh2. In HCs, suppressing glycolysis similarly decreased the activation of both cTfh2 and cTfh17 cells, as well as their capacity to activate B cells.

In patients with SLE, an increased frequency of CXCR5^+^ICOS^+^ cTfh cells corroborated the abnormal immune profile in lupus (33). Although the overall frequencies of cTfh cells and subsets (cTfh1, cTfh2, cTfh17) were similar between patients with SLE and HCs, the elevated ICOS^+^PD-1^+^ cTfh levels across all subsets in patients with SLE suggest heightened activation. This increased activation likely contributes to the pathogenic role of cTfh cells in SLE by promoting excessive B cell help, germinal center reactions, and autoantibody production, which are central to lupus pathogenesis (34).

Metabolic profiling revealed significant differences in glycolytic activity among cTfh subsets. TCR-mediated stimulation increased glucose uptake in all cTfh subsets from both HCs and patients with SLE. Surprisingly, glucose uptake was higher in cTfh cells from HCs than in those from patients with SLE across all subsets, despite similar glycolytic capacity between the 2 groups. This observation aligns with the finding that mTORC1 activity did not differ significantly between cTfh subsets from HCs and patients with SLE. These data do not support a previous study reporting increased glycolysis and mitochondrial metabolism in total CD4^+^ T cells from patients with SLE compared with HCs (16). This discrepancy could be attributed to differences in cell types (total CD4^+^ T cells versus cTfh cells) or in treatment regimens. While hydroxychloroquine was commonly used in both studies, our cohort included more patients treated with immunosuppressants such as mycophenolate mofetil, known to suppress cytokine production and cell proliferation (35).

Despite similar glucose uptake levels across activated cTfh subsets, Seahorse assays revealed higher glycolysis and glycolytic capacity in cTfh17 cells compared with other subsets during activation. While increased *GLUT1* mRNA expression was not consistently observed following stimulation, GLUT1 protein levels were upregulated. It is plausible that the *GLUT1* mRNA levels, which may initially increase upon stimulation, return to baseline levels after overnight stimulation. Given previous reports of increased GLUT1 expression in Th17 cells from patients with SLE (36), this increased GLUT1 protein may contribute to the observed enhanced glycolysis. Due to limited cell numbers, we were unable to comprehensively compare GLUT1 protein expression levels between cTfh subsets. Further investigation, including quantitative comparison of GLUT1 expression in cTfh subsets between HCs and patients with SLE, is needed to elucidate the lower glucose uptake observed in SLE cTfh cells. Given reports of mTORC1 activity increasing GLUT1 expression in murine Tfh-like cells (18), it is possible that cTfh17 cells, which exhibit higher mTORC1 activity, also express higher levels of GLUT1 protein compared with other subsets.

Gene expression analysis further illustrated the metabolic landscape: Activated cTfh1 and cTfh17 cells showed upregulated glycolysis-related genes (*HIF1A*, *GAPDH*, *IRF4*, and *ENO1*), with *ENO1* particularly elevated in cTfh17 cells, highlighting their preferential engagement of glycolysis. This enhanced glycolytic activity is functionally relevant, as cTfh17 cells supported higher B cell activation and differentiation, as previously observed by Morita et al. (10). Notably, SLE-driven cTfh17 cells promoted greater plasmablast differentiation and immunoglobulin production than those from HCs. Gao et al. recently demonstrated that Tfh17 cells are superior in supporting memory B cell activation compared with Tfh1 and Tfh2 in both humans and mice (37), underscoring the pathogenic potential of Tfh17 in lupus.

The dependency of cTfh17 cells on glucose metabolism was further demonstrated by glucose deprivation experiments. Under glucose-free conditions, cTfh17 cells exhibited reduced expression of activation markers (CD69, ICOS, PD-1) and pro-inflammatory cytokines (IL-17A, IL-10, IL-2, GM-CSF), indicating the necessity of glucose metabolism for their activation and effector functions. Glucose restriction reduced costimulatory molecule upregulation in cTfh2 cells but did not affect cytokine production, suggesting that while glycolysis is essential for costimulatory molecule expression in both cTfh subsets, it specifically affects cytokine production only in cTfh17 cells from patients with SLE. In contrast, glucose deprivation had broader effects on both costimulatory molecules and cytokine production in both cTfh subsets from HCs. This selective impact of glucose metabolism on SLE cTfh subsets suggests the potential involvement of alternative metabolic regulation or epigenetic modifications affecting cytokine expression in cTfh cells from patients with SLE. Targeting glucose metabolism may therefore offer a selective therapeutic strategy to modulate cTfh17 activity in SLE.

Pharmacological inhibition studies further verified the critical role of glucose metabolism. Inhibition of glycolysis with STF-31 reduced both the activation and effector function of cTfh17 cells, leading to diminished B cell activation. Importantly, despite similar glycolysis levels between HCs and patients with SLE, IL-21 production (a key effector cytokine of cTfh cells) responded to glucose deprivation only in HCs but not in patients with SLE. This suggests the presence of an alternative, GLUT1- and glycolysis-independent metabolic pathway for IL-21 production in patients with SLE or potentially cell-intrinsic differences in cTfh17 cells between these groups.

Our findings emphasize the importance of considering metabolic dependencies in therapeutic development. The distinct metabolic profiles of cTfh subsets, particularly the glycolytic emphasis in cTfh17 cells, suggest that metabolic reprogramming affects their function. Targeting glycolysis may selectively inhibit pathogenic cTfh17 cells while sparing other immune cells, potentially reducing the risk of broad immunosuppression. This approach could be particularly beneficial for lupus patients with an expanded cTfh17 populations. Targeting glucose metabolism alone may not fully address the metabolic requirements of other cTfh subsets, underscoring the need for further exploration. The results of the p-S6 assay suggest that mTORC1 activity is enhanced in activated cTfh17 cells compared with other cTfh subsets in patients with SLE. Given mTORC1’s role in regulating glycolysis, this enhanced activity may explain the observed metabolic differences between cTfh17 and other cTfh subsets. Future studies should investigate the molecular mechanisms of metabolic reprogramming in cTfh17 cells, particularly concerning cytokine production, and find the metabolic dependencies of cTfh1 and cTfh2 in SLE. Investigating upstream regulators of enhanced glycolysis in these cells could uncover additional therapeutic targets. Moreover, evaluating the metabolic needs of cTfh cells across SLE progression and treatment responses could offer insight into adaptive metabolic changes in the disease. In vivo efficacy and safety studies of metabolic inhibitors in preclinical SLE models are also essential. Understanding long-term effects on immune function and disease progression is critical for translating these findings into clinical applications. One limitation of this study is that the observed differences were detected only under strong stimulation conditions. Further investigation is warranted to determine whether these metabolic differences are also present under more physiological conditions, such as stimulation with antigen-presenting cells loaded with antigenic peptides.

In summary, our study highlights shared and unique metabolic dependencies among cTfh subsets, underscoring the pivotal role of glucose metabolism in the activation and function of cTfh17 cells in both patients with SLE and HCs. The heightened glycolytic capacity of cTfh17 cells supports their pathogenic role, and targeting glucose metabolism may present a promising therapeutic strategy for modulating cTfh17-mediated immune responses in SLE. While suppressing glycolysis similarly reduces the activation and B cell–activating capacity of both cTfh2 and cTfh17 cells in HCs, this effect is more selective in cTfh17 cells from patients with SLE, suggesting the involvement of additional regulatory mechanisms or pathways interacting with glycolysis in SLE that enhances cTfh17 glucose dependence. These findings lay the groundwork for future research into metabolic intervention, potentially leading to improved clinical outcomes for patients with SLE.

## Methods

### Sex as a biological variable.

Our study included only females for both patients with SLE and healthy individuals because of strong female bias in SLE.

### Human samples.

Lupus patient samples were collected through the rheumatology clinic at Northwell Health. Samples from healthy individuals were provided by the New York Blood Center. Only samples from female individuals under the age of 55 were included in the study. Demographic information with clinical features of patients with SLE is provided in Table 1.

### Isolation and stimulation of lymphocytes from PBMCs.

Blood samples were processed immediately after collection, and PBMCs were isolated using Ficoll density gradient centrifugation. Briefly, whole blood was diluted with an equal volume of HBSS, layered onto Ficoll-Paque, and centrifuged at 800*g* for 20 minutes at 20°C. The leukocyte layer was collected, transferred to a new tube, and washed twice with HBSS. For the isolation of cTfh subsets and memory B cells, PBMCs were stained with surface markers, and each population was sorted by cell sorter. Following sorting, cTfh subsets were cultured in medium alone (RPMI1640 [Gibco] with 10% heat-inactivated FBS [Gibco], 1× penicillin/streptomycin, 2 mM glutamine, and 0.05 mM of β-mercaptoethanol) or activated with anti-CD2/CD3/CD28 activation beads (cells to bead ratio of 1:1) (human T Cell Activation/Expansion Kit, catalog 130-091-441; Miltenyi Biotec) for 16 hours in a 37°C incubator. For the inhibitor assay, cTfh17 and cTfh2 cells were treated with STF-31 (20 μM; catalog SML 11045​; Sigma-Aldrich; Merck KGaA), IACS-010759 (10 nM; catalog HY-112037; MedChemExpress), or C968 (5 μM; catalog HY-12682; MedChemExpress) during activation. To test glucose dependency, cells were activated in a glucose-free medium (RPMI1640 medium, no glucose [Gibco], supplemented with 10% dialyzed FBS [Gibco],1% penicillin/streptomycin, 2 mM glutamine, and 0.05 mM of β-mercaptoethanol).

### Flow cytometry analysis and antibodies for flow cytometry.

Surface staining was performed by incubating the cells with antibodies and FVD eFluor 506 (Invitrogen) in staining buffer (HBSS with 2% FBS and 1 mM EDTA) on ice for 15 minutes, protected from light. After incubation, cells were washed twice with staining buffer and proceeded to flow cytometry analysis. Intracellular staining was conducted following surface staining, using the FOXP3 staining buffer kit (eBioscience) according to the manufacturer’s protocol. Measurement of phosphorylation level of S6 was performed using Phosflow Lyse/Fix buffer (BD Biosciences) and Perm Buffer III (BD Biosciences) according to the manufacturer’s protocol. Stained cells were read on a BD Biosciences LSRFortessa X-20 Cell Analyzer, and the data were analyzed with FlowJo software (Tree Star).

### Antibodies.

FITC anti-human CD69 (clone: FN50), PE/Dazzle 594 anti-human/mouse/rat CD278 (ICOS) (clone C398.4A), PerCP/Cy5.5 anti-human CD183 (CXCR3) (clone G025H7), APC-Cyanine 7 anti-human CD4 (clone RPA-T4), PE/Cy7 anti-human CD196 (CCR6) (clone G034E3), PE anti-human CD185 (CXCR5) (clone J252D4), PE/Cy7 anti-human CD19 (clone HIB19), PE anti-human CD27 (clone M-T271), PerCP anti–HLA-DR (clone L243), APC anti-human CD86 (clone IT2.2), BV421 anti-human PD-1 (clone EH12.2H7), FITC anti-human CD45RA (clone HI100), PerCP/Cy5.5 anti-human IgM (clone MHM-88), and PerCP/Cy5.5 anti-human IRF4 (clone IRF4.3E4) were purchased from BioLegend. PE-eFluor 610 anti-human CD38 (clone HIT2) was purchased from Invitrogen. FITC anti-human IgG (clone G18-145) was purchased from BD Pharmingen, and APC anti-human IgA (clone IS11-8E10) was purchased from MACS-Miltenyi. AF488 anti–p-S6 (Ser235/236) Alexa Fluor 488 conjugate (clone D57.2.2E) was purchased from Cell Signaling Technology.

### In vitro differentiation of PCs.

To assess B cell activation and differentiation by cTfh subsets, memory B cells (FSC^lo^CD19^+^CD27^+^) were cocultured with autologous cTfh2 or cTfh17 cells at a 1:2 (T/B) ratio for 5–6 days. Memory B cells were cultured in medium alone as a negative control or in a differentiation cocktail (50 U/mL of IL-2, 250 ng/mL of IL-10, 50 ng/mL of IL-21, and 2.5 μmol of CpG ODN2006) as a positive control. After culture, PCs were identified by high levels of CD27 and CD38 expression, and PC isotypes were determined by intracellular immunoglobulin staining. In some experiments, cTfh17 cells were preincubated with metabolic inhibitors overnight before coculture with memory B cells.

### Measurement of cytokine or immunoglobulin from the culture.

To measure cytokines, cTfh2 and cTfh17 cells were isolated and stimulated with anti-CD2/3/28 activation beads (cell/bead ratio is 1:1) in complete medium or glucose-free media (1 × 10^6^/mL). Supernatants were collected, and cytokines were measured using a U-plex human biomarker group 1 multiplex assay (MSD). Immunoglobulin secretion was measured from the 5-day culture of memory B cells and cTfh subsets using immunoglobulin ELISA. Briefly, purified anti-human immunoglobulin capture antibodies (10 μg/mL in PBS) were coated onto a Costar 96-well assay plate (Corning) overnight at 4°C. The following day, the plate was washed and incubated with blocking buffer (10% BSA in PBS) for 1 hour at room temperature. After blocking, purified immunoglobulin standards or supernatants (diluted in 0.2% BSA in PBS) were added and incubated for 2 hours at room temperature. AP-conjugated anti-immunoglobulin antibodies (1 μg/mL in 0.2% BSA in PBS) were added and incubated for 1 hour at room temperature. AP substrate (MilliporeSigma) was prepared in substrate buffer (50 mM NaHCO_3_ and 1 mM MgCl_2_) and used to develop the assay for 30 minutes. OD was measured using a Victor3 plate reader (PerkinElmer). Antibodies and standards for immunoglobulin ELISA were obtained from Southern Biotech: goat anti-human IgG-UNLB (catalog 2040-01), goat anti-human IgG-AP (catalog 2040-04), goat anti-human IgA-UNLB (catalog 2050-01), goat anti-human IgA-AP (catalog 2050-04), goat anti-human IgM-UNLB (catalog 2020-01), goat anti-human IgM-AP (catalog 2020-04), human IgG (catalog 0150-01), human IgA kappa (catalog 0155K-01), and human IgM lambda (catalog 0158L-01).

### Metabolic measurement.

Respiratory capacity and glycolytic capacity of cTfh subsets were measured using the Agilent Seahorse XFp mini-Analyzer, following the manufacturer’s protocol. Briefly, isolated cTfh subsets were activated with anti-CD2/3/28 activation beads at 1:1 ratio for 16 hours. A total of 2 × 10^5^ cells were plated per well, and ECAR and OCR were measured using the XFp Mito stress test kit (103010-100) or XFp Glycolysis stress test kit (103017-100) (Agilent). The assay medium was prepared using nonbuffered RPMI medium (Agilent) supplemented with 2.5 μM glucose, 2 mM glutamine, and 1 μM pyruvate (for Mito stress assay) or with 2 mM glutamine and 1 μM pyruvate (for glycolysis assay). Experiments were performed in 3 technical replicates and a minimum of 3 biological replicates. Data analysis was conducted using Agilent’s analysis software provided on their website.

To measure the mitochondrial membrane potential, ROS production, and glucose uptake, cTfh subsets were isolated and cultured at 1 × 10^6^/mL in medium alone or with activation beads for 16 hours. After activation, cells were stained with 5 nM MitoTracker Green (M7514) (Invitrogen), 5 nM MitoTracker Red (M22425) (Invitrogen), 1 μM MitoSOX (M36008) (Invitrogen), or 100 μM 2-NBDG (Calbiochem) for 20 minutes. Following incubation, cells were washed with 10 times the volume of staining buffer, and fluorescence of each dye was measured by flow cytometry. Extracellular lactate production was measured using the Lactate Colorimetric/Fluorometric assay kit (Biovision).

### RNA isolation and qRT-PCR.

Sorted cTfh subsets were cultured overnight in medium alone or with anti-CD2/3/28 to activate cells for activation. After culture, cells were washed once with ice-cold HBSS, and total RNA was extracted using the RNA extraction kit from Zymo Research. A 200 μg of total RNA was reverse-transcribed to cDNA using iScript cDNA synthesis kit (Bio-Rad). qRT-PCR was performed using the Taqman qPCR master mix (Invitrogen) with gene-specific primers purchased from TaqMan (Invitrogen). PCR was conducted on a LightCycler 480 II (Roche) with the following Taqman primer sets: *POLR2A* (Hs00172187_m1), *IRF4* (Hs00180031_m1), *MTOR* (Hs00234508_m1), *SLC1A5* (Hs01056542_m1), *GLUD1* (Hs03989560_s1), *GLS* (Hs01014020_m1), *ACACA* (Hs01046047_m1), *FABP5* (Hs02339439_g1), *NMT1* (Hs00221506_m1), *MT-ND1* (Hs02596873_s1), *SDHC* (Hs01698067_s1), *SLC2A1* (Hs00892681_m1), *HIF1A* (Hs00153153_m1), *GAPDH* (Hs0275899_g1), *ENO1* (Hs00361415_m1), *LDHB* (Hs00929956_m1), *ATP5A1* (Hs00900735_m1), *SDHA* (Hs00188166_m1), and *UQCRFS1* (Hs04194251_g1). Ct values for each gene were normalized to the housekeeping gene *POLR2A* to calculate relative expression levels.

### Western blotting.

Sorted cTfh subsets were cultured overnight in medium alone or with anti-CD2/3/28 to activate cells. After culture, cells were washed once with ice-cold PBS and lysed using Pierce RIPA buffer (Thermo Fisher Scientific) containing protease inhibitor cocktail (Thermo Fisher Scientific) and phosphatase inhibitor cocktail (Thermo Fisher Scientific). Samples were incubated for 30 minutes on ice and centrifuged at 4°C, 21,000*g*, for 20 minutes, and supernatants were collected. Samples were denatured and reduced by overnight incubation at 4°C in 4× Laemmli Sample Buffer (catalog 1610747, Bio-Rad) containing 50 mM DTT. The samples were then electrophoresed on 10% SDS-PAGE, transferred to PVDF membranes, and incubated in 5% BSA in TBS-Tween for 1 hour at room temperature. The membranes were then incubated for 16 hours at room temperature with a 1:250 dilution of anti-GLUT1 antibody (catalog 12939, D3J3A, Cell Signaling Technology) in 5% BSA or 1:2,000 dilution of anti-β-actin antibody (catalog sc-47778, C4, Santa Cruz Biotechnology) in 5% BSA. After incubation with primary antibodies, the membranes were incubated with anti-rabbit or anti-mouse IgG horseradish peroxidase–conjugated secondary antibodies (catalog 1705046, catalog 1705047, Bio-Rad) for 1 hour at room temperature. Immunoreactivity was detected using the Sapphire biomolecular imager (Azure Biosystems).

### Statistics.

Data were not assumed to be Gaussian distributed; therefore, comparisons between 2 groups were performed using the Wilcoxon paired test, and multiple comparisons were conducted using a 1-way ANOVA with correction as specified in this paper. Statistical analysis was performed using Prism 9.0 software (GraphPad). A *P* value less than 0.05 was considered significant.

### Study approval.

The study was approved by the Institutional Review Board of The Feinstein Institutes for Medical Research (approval number: 17-0075). Written informed consent was obtained from all participants and/or their parents/guardians.

### Data availability.

The raw data are reported in the Supporting Data Values file. All the data and protocols will be distributed from the corresponding author upon request.

## Author contributions

VK and TM conducted experiments, acquired and analyzed data, and contributed to manuscript preparation. HT also performed the experiments. CA and MM provided patient samples and analyzed demographic information. SJK conceived the study, designed the experiments, analyzed data, and contributed to manuscript preparation. The order of co–first authors reflects their relative contribution to the experimental work.

## Supplementary Material

Supplemental data

Unedited blot and gel images

Supporting data values

## Figures and Tables

**Table 1:**
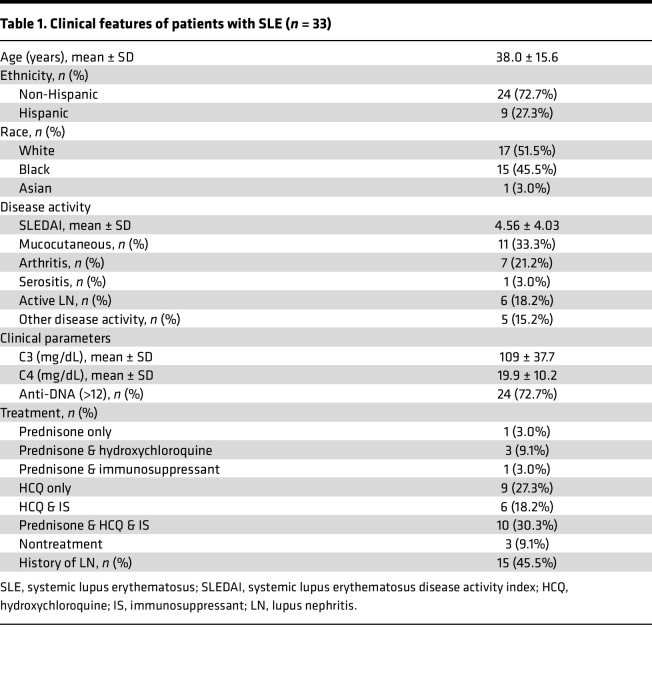
Clinical features of patients with SLE (*n* = 33)

## Version 1 07/22/2025

Electronic publication
