# Supplementary material for: Metabolic pathways within cTfh subsets and glucose-dependent activation of cTfh17 in SLE and healthy individuals
Source: JCI Insight. 2025 Jul 22;10(14):e189858. doi: 10.1172/jci.insight.189858 (PMC12288977; doi:10.1172/jci.insight.189858)
Supplement: Supplemental data [file jciinsight-10-189858-s018.pdf]

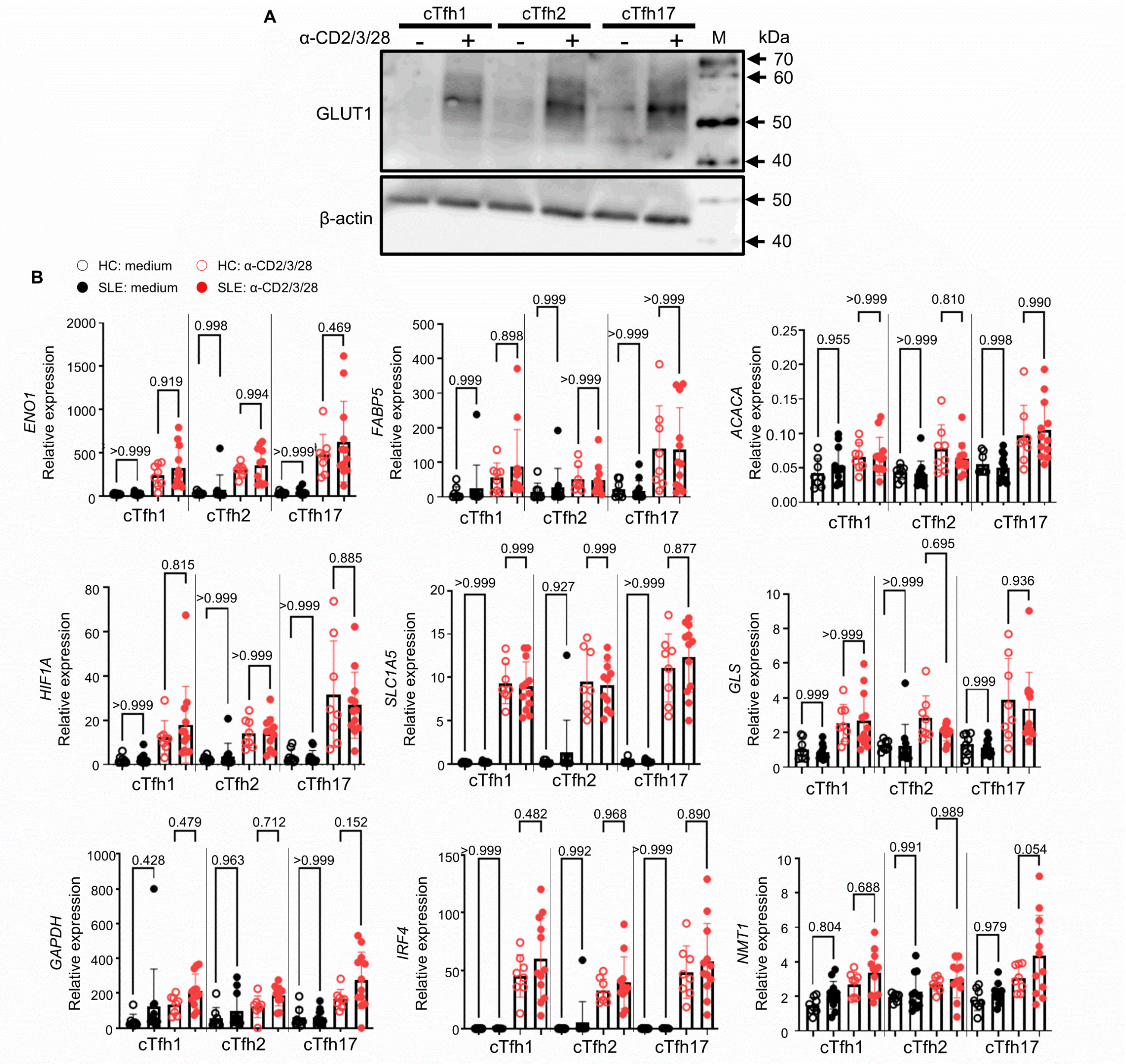

Supplemental Figure 1: Metabolic gene expression in cTfh subsets between HCs and SLE patients. **(A)** cTfh subsets were isolated from HCs and stimulated with anti-CD2/3/28 beads for 16 h, then GLUT1 protein expression was assessed by western blotting. A representative image from two independent experiments. **(B)** Gene expression levels were measured from cTfh subsets isolated from HCs (black circles) and SLE patients (red circles), cultured overnight alone (open circles) or with stimulation beads (closed circles). After culture, total RNA was extracted, gene expression was measured by qRT-PCR. Relative expression was calculated using the housekeeping gene *POLR2A* as a reference (n=9). Each dot represents an individual sample mean ± SD. Statistical analysis performed by one-way ANOVA with Bonferroni's post-test correction.

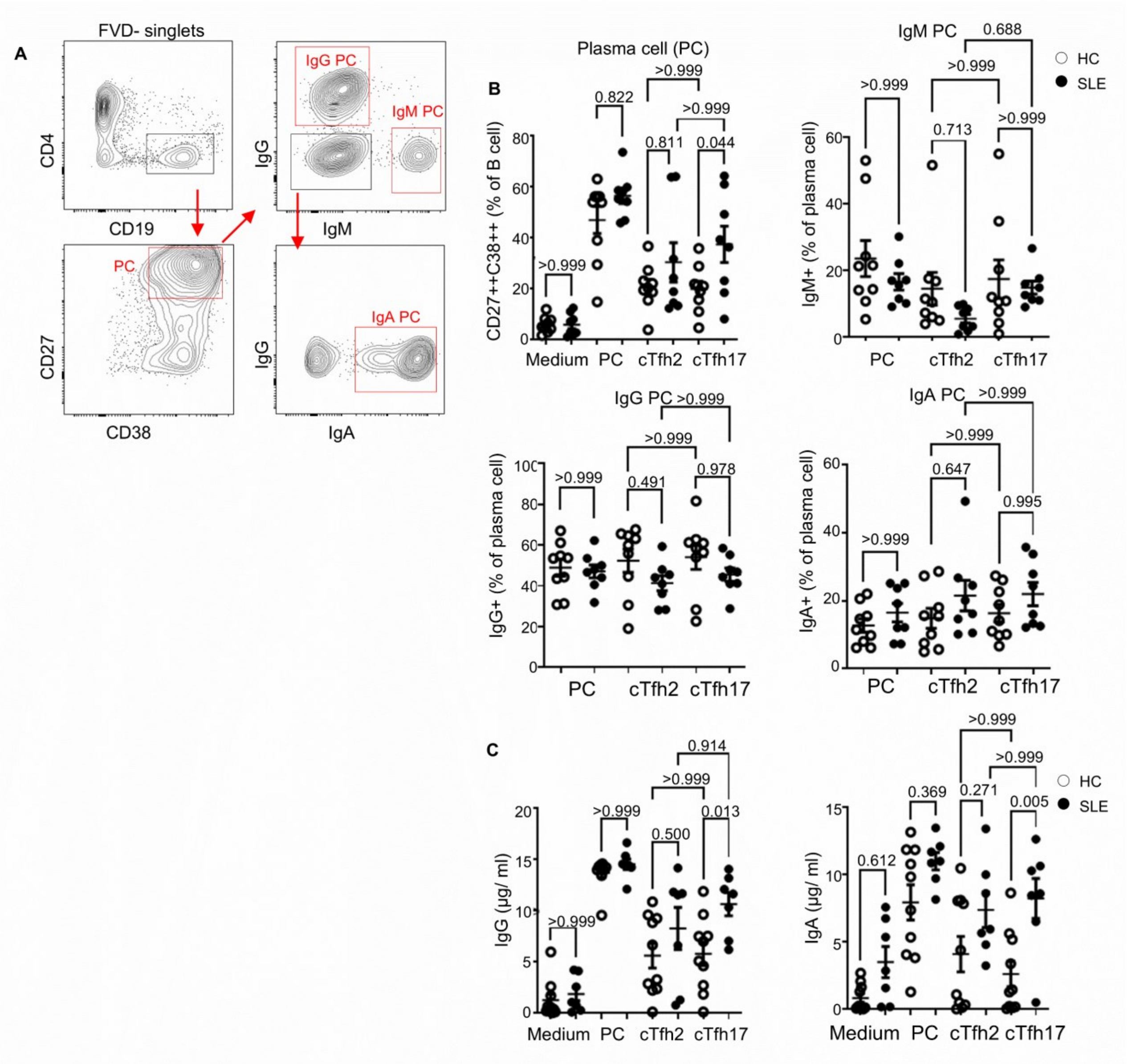

Supplemental Figure 2: Increased PC differentiation from memory B cells by SLE cTfh17 cells. Memory B cells ($CD27^{+}CD19^{+}$) and cTfh subsets (cTfh2 and cTfh17) were isolated from the same individual and co-cultured for 5 days. Memory B cells alone served as a negative control, while memory B cells cultured with PC differentiation cocktail (CpG ODN, IL-2, IL-10, and IL-21) served as a positive control. **(A)** Representative flow cytometry image showing PCs ($CD27^{++}CD38^{++}$) and PC isotypes. **(B)** Total PCs were quantified from FVD- live B cells, and IgM^{+}, IgG^{+}, and IgA^{+} PC were quantified from total PCs and graphed. **(C)** Secreted IgG and IgA in the culture supernatant were measured by ELISA. Open circles represent HC samples, and closed circles represent SLE samples (n=9). Each dot represents an individual sample, with bars indicating the mean ± SE. Statistical analysis was performed using ordinary one-way ANOVA with Bonferroni's post-test correction.

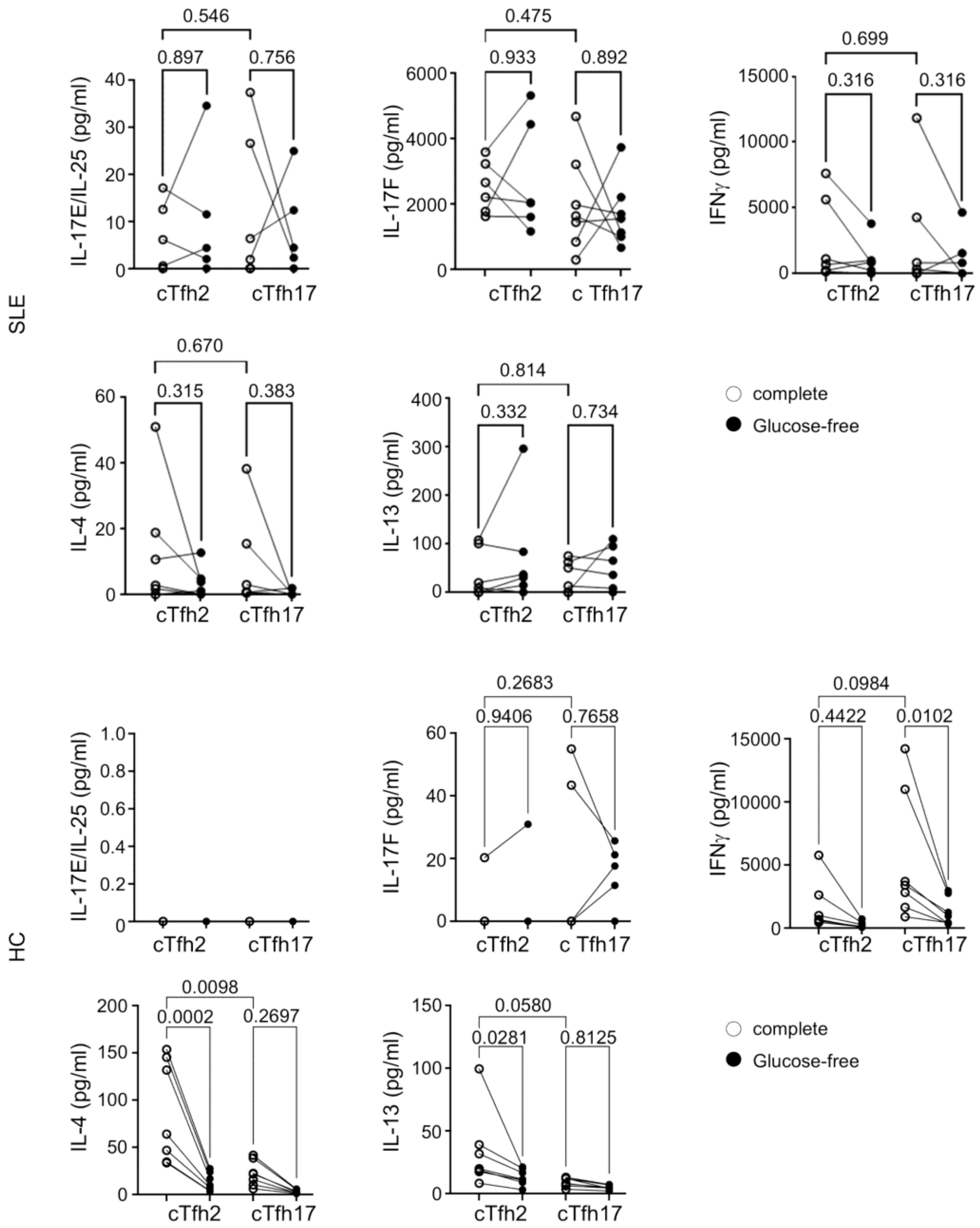

Supplemental Figure 3: Glucose requirement for Th17 and Th2 cytokines and IFNγ production. cTfh2 and cTfh17 cells were isolated from SLE patients and HCs and cultured overnight with or without anti-CD2/3/28 beads in either complete or glucose-free medium. Cytokine levels in the collected supernatants were then measured using an MSD multiplex assay. Data from SLE patients are shown on the left, and data from HCs are shown on the right. Open circles represent complete medium, and closed circles represent glucose-free conditions (n=7). Each dot represents an individual sample. Statistical analysis was performed using ANOVA with Sidak's multiple comparison test.

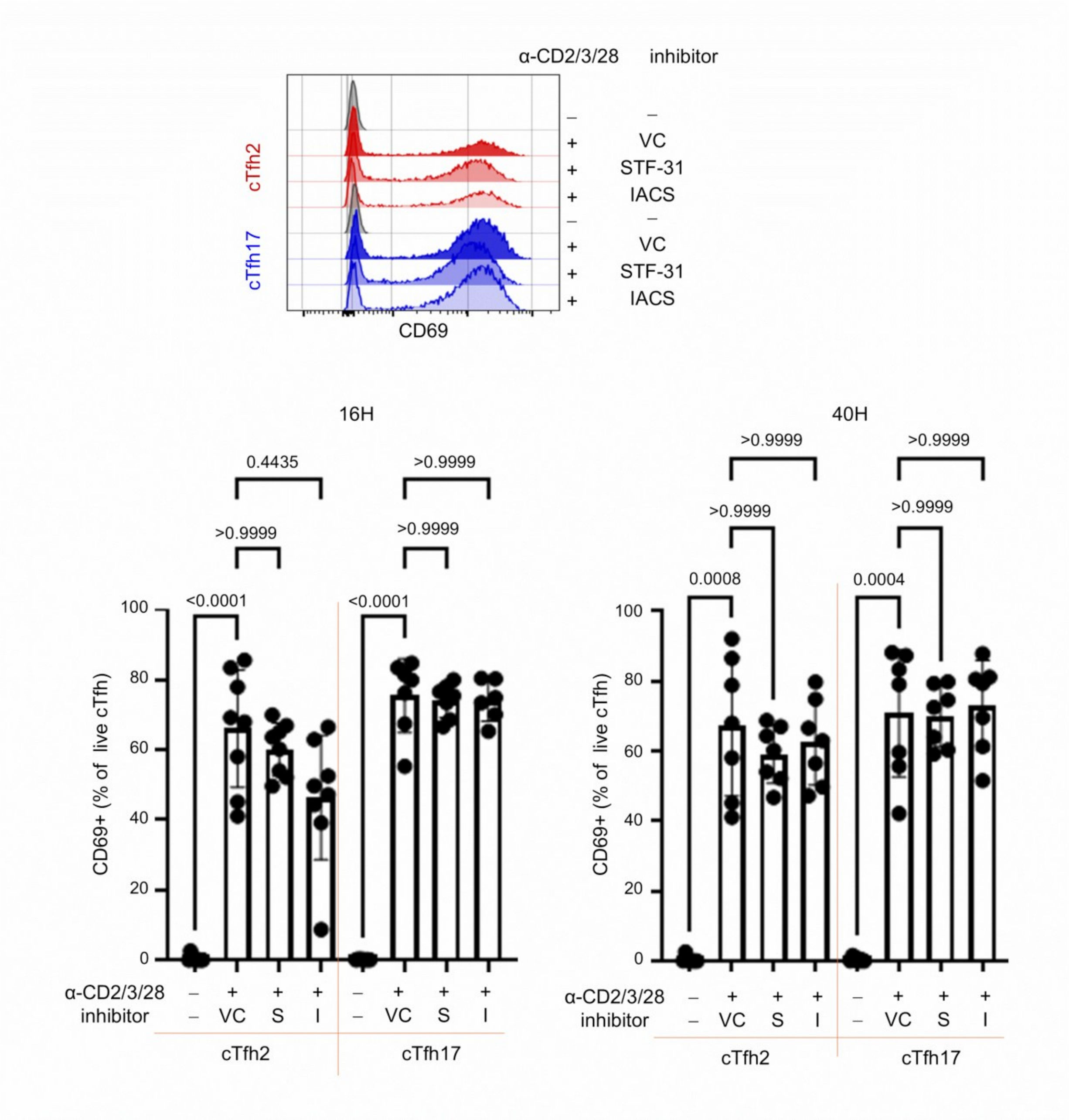

Supplemental Figure 4: STF-31 or IACS-010759 pre-treatment does not impact CD69 expression in activated cTfh 2 and cTfh17 cells. cTfh2 and cTfh17 cells were isolated from SLE patients and activated with or without activation beads under various inhibitor conditions (VC: DMSO vehicle control, S: STF-31, and I: IACS-010759). Representative overlaid histogram of CD69 expression is on the top row. The frequency of $CD69^+$ cTfh2 and cTfh17 cells was quantified from viable cells and graphed at the indicated time point (bottom row) (n=6). Each dot represents an individual sample mean ± SD. Statistical analysis performed by ordinary one-way ANOVA with Bonferroni's post-test correction.

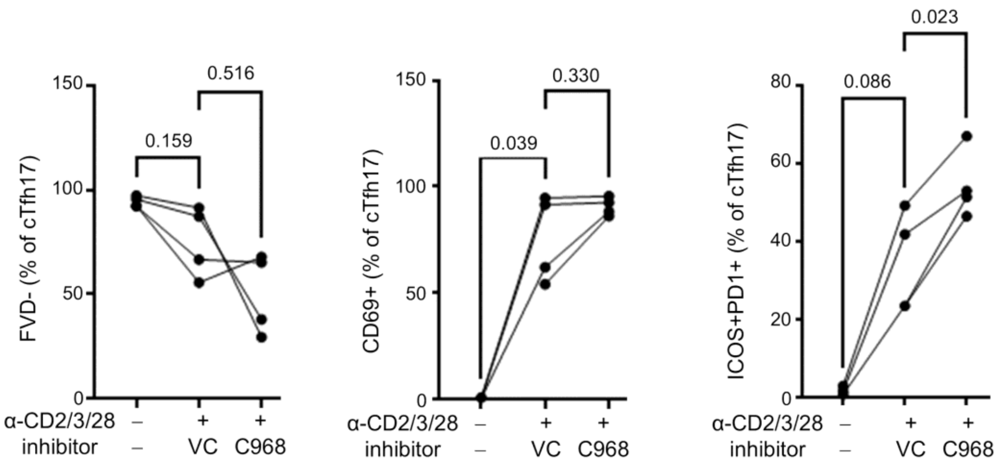

Supplemental Figure 5: C968, a glutaminase inhibitor, pre-treatment does not suppress activation of cTfh17 cells. cTfh17 cells were isolated from SLE patients and cultured in medium alone or with activation beads with VC (DMSO) or C968. The viability was assessed and the percentage of $CD69^{+}$ or $ICOS^{+}PD1^{+}$ was quantified from live cTfh17 cells and graphed (n=4). Each dot represents an individual sample, with statistical analysis performed by ordinary one-way ANOVA with Bonferroni's post-test correction.
