# Supplementary material for: Metabolic pathways within cTfh subsets and glucose-dependent activation of cTfh17 in SLE and healthy individuals
Source: JCI Insight. 2025 Jul 22;10(14):e189858. doi: 10.1172/jci.insight.189858 (PMC12288977; doi:10.1172/jci.insight.189858)
Supplement: Unedited blot and gel images [file jciinsight-10-189858-s019.pdf]

Uncropped/unedited blot image for Supplemental Figure 1A.
The red rectangle indicate the region has been used for Supplemental Figure 1A.

GLUT1

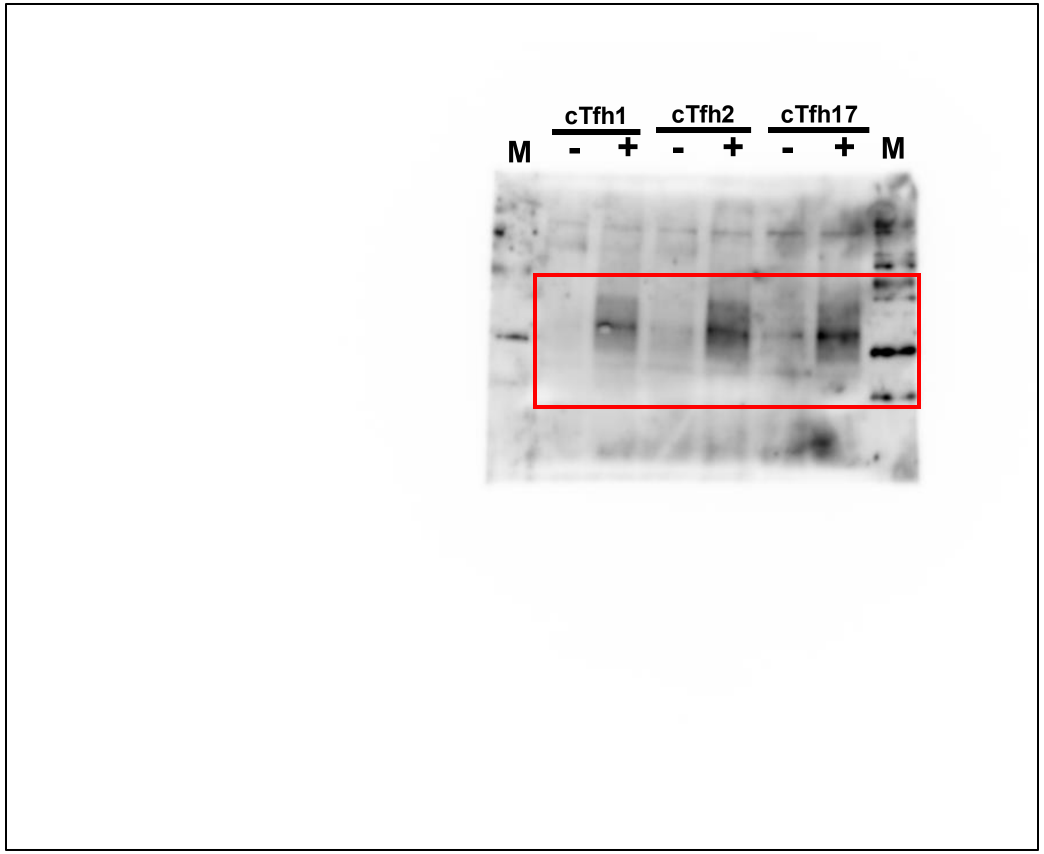

β-actin

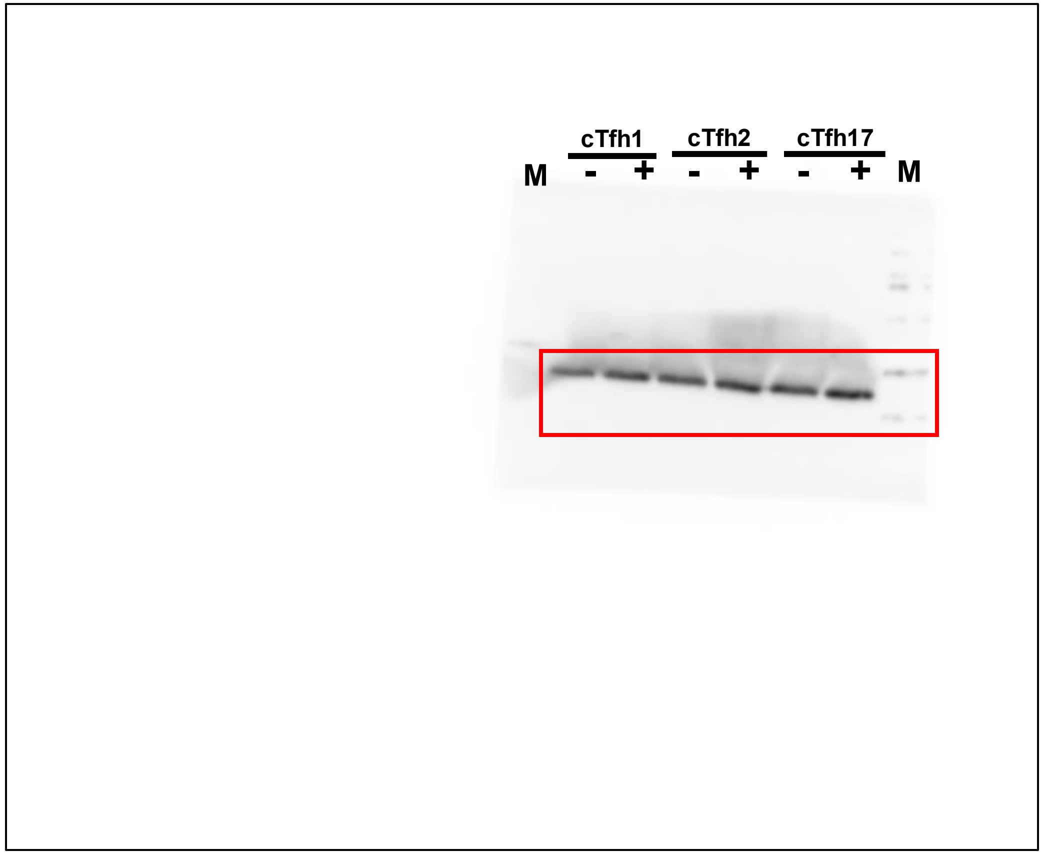

-, unstimulated
+, stimulated
M, marker
anti-GLUT1 antibody (cat. no.12939, D3J3A, Cell Signaling Technology)
anti-β-actin antibody (cat. No.sc-47778, C4, Santa Cruz Biotechnology)
